# The PTK7 and ROR2 Protein Receptors Interact in the Vertebrate WNT/Planar Cell Polarity (PCP) Pathway*

**DOI:** 10.1074/jbc.M115.697615

**Published:** 2015-10-23

**Authors:** Sébastien Martinez, Pierluigi Scerbo, Marilyn Giordano, Avais M. Daulat, Anne-Catherine Lhoumeau, Virginie Thomé, Laurent Kodjabachian, Jean-Paul Borg

**Affiliations:** From the ‡ CRCM, Cell Polarity, Cell Signaling, and Cancer “Equipe Labellisée Ligue Contre le Cancer”, INSERM, U1068, 13009 Marseille, France,; the § Institut Paoli-Calmettes, 13009 Marseille, France,; the ¶ Aix-Marseille Université, 13284 Marseille, France,; the ‖ CNRS, UMR7258, 13009 Marseille, France, and; the ** Institut de Biologie du Développement de Marseille, Aix-Marseille Université, CNRS, 13288 Marseille, France

**Keywords:** JNK, cell polarity, development, protein-tyrosine kinase (tyrosine kinase), Wnt pathway, JNK, PTK7, planar cell polarity, ROR2, WNT5A

## Abstract

The non-canonical WNT/planar cell polarity (WNT/PCP) pathway plays important roles in morphogenetic processes in vertebrates. Among WNT/PCP components, protein tyrosine kinase 7 (PTK7) is a tyrosine kinase receptor with poorly defined functions lacking catalytic activity. Here we show that PTK7 associates with receptor tyrosine kinase-like orphan receptor 2 (ROR2) to form a heterodimeric complex in mammalian cells. We demonstrate that PTK7 and ROR2 physically and functionally interact with the non-canonical WNT5A ligand, leading to JNK activation and cell movements. In the *Xenopus* embryo, Ptk7 functionally interacts with Ror2 to regulate protocadherin *papc* expression and morphogenesis. Furthermore, we show that Ptk7 is required for *papc* activation induced by Wnt5a. Interestingly, we find that Wnt5a stimulates the release of the tagged Ptk7 intracellular domain, which can translocate into the nucleus and activate *papc* expression. This study reveals novel molecular mechanisms of action of PTK7 in non-canonical WNT/PCP signaling that may promote cell and tissue movements.

## Teaser

**Background:** The planar cell polarity pathway plays important roles in morphogenetic processes.

**Results:** PTK7 and ROR2 form a heterodimeric complex and bind to WNT5A, promoting JNK phosphorylation and regulating expression of paraxial protocadherin.

**Conclusion:** PTK7 and ROR2 promote cell movement in mammalian cells and coordinate cell polarity during morphogenetic movements.

**Significance:** We reveal new mechanisms of action of PTK7 in WNT/PCP signaling.

## Introduction

Initially described in *Drosophila melanogaster*, planar cell polarity (PCP)^5^ regulates multiple processes during embryonic development and tissue homeostasis. Its importance in development is best highlighted by its role in convergence extension cell movements during gastrulation that are necessary for the proper elongation of the anterior-posterior body axis. PCP serves also to orient apical structures or groups of cells within the plane of the epithelium and drives oriented cell migration of epithelial and non-epithelial cell types (1–4). PCP is assigned molecularly to a non-canonical WNT pathway (hereafter named the WNT/PCP pathway) that, in contrast to the canonical WNT pathway, does not involve the transcriptional regulator β-catenin. WNT/PCP utilizes small Rho-like GTPases and JNK to promote actin cytoskeleton reorganization and cellular movements (5). Several WNT/PCP genes have been isolated from various species and have been shown to encode conserved proteins across evolution at the molecular and functional levels (6, 7). A striking feature of WNT/PCP signaling is the implication of a large spectrum of cell surface receptors belonging to various protein families, including the multipass membrane (Fz3, Fz6, VANGL1, VANGL2, and CELSR1), proto-cadherin (FAT4 and PAPC), and tyrosine kinase receptor (PTK7, ROR2, and RYK) families, that can interact directly or indirectly with WNT ligands (8). How these receptors cross-talk at the plasma membrane and how they initiate downstream molecular cascades remain largely open questions.

PTK7 is a tyrosine kinase receptor (RTK) implicated in the WNT/PCP pathway in mice, zebrafish, and *Xenopus. Ptk7*-deficient mice die perinatally because of severe embryonic defects of PCP and convergent extension. Embryos have an impaired gastrulation, misoriented stereociliary bundles of sensory hair cells in the inner ear, defective neural tube closure, smaller kidneys, eyelid closure defects, and polydactyly. Knockdown of *ptk7* in *Xenopus* leads to PCP-like phenotypes, including neural tube closure defects and incomplete blastopore closure (9–14). At the structural level, PTK7 is well conserved across evolution and displays a classical molecular organization with an extracellular region comprising seven extracellular immunoglobulin loops, a transmembrane region, and an inactive intracellular tyrosine kinase domain able to translocate into the nucleus upon proteolytic cleavage (15–18). Both extra- and intracellular domains of PTK7 are required for its functions in mammals, zebrafish, and *Xenopus* (9, 10, 13). Previous works have detected interaction between PTK7 and cell surface receptors unrelated to the WNT/PCP pathway (VEGFR1, Plexin-A, and LRP6) (19–21). In addition, PTK7 has been shown to co-immunoprecipitate with Fz7 and canonical WNT ligands (WNT3 and WNT8) to repress canonical WNT signaling in *Xenopus* (11), whereas it binds WNT2 and WNT4 in *Drosophila* to trigger non-WNT/PCP-related functions (11, 22). Overall, how PTK7 transduces a WNT/PCP signaling cascade from the plasma membrane remains largely unknown.

In analogy to poorly active RTKs that heterodimerize with heterologous active RTKs to transmit a signal (23), we hypothesized that PTK7 may utilize such a means to propagate WNT/PCP functions. We focused on ROR2, a catalytically active RTK that, upon binding to non-canonical WNT5A, triggers WNT/PCP functions in *Xenopus* and in the mouse (24). We find that PTK7 and ROR2 form a heterodimeric complex and that PTK7, like ROR2, binds to WNT5A and promotes JNK phosphorylation and cell movements in mammalian cells. In *Xenopus*, Ptk7 and Ror2 interact functionally and regulate the expression of paraxial protocadherin (*papc*), a gene that coordinates the polarity of cells during morphogenetic movements (25, 26). Furthermore, we report that, in *Xenopus*, Wnt5a triggers the release and translocation of the Ptk7 intracellular domain in the nucleus, where it can activate *papc* expression. This study highlights some new mechanisms used by PTK7 to mediate WNT/PCP signaling in vertebrates.

## Experimental Procedures

### 

#### 

##### Cell Culture and Cell Transfection

HEK 293T cells were purchased and grown in accordance with ATCC recommendations. Cells were grown in DMEM supplemented with 100 units/ml of penicillin and 100 mg/ml of streptomycin. MEFs isolated from WT or gene-trapped *ptk7* (PTK7 KO) mice (9) were grown in DMEM supplemented with 100 units/ml of penicillin, 100 mg/ml of streptomycin, 1 mm sodium pyruvate, 1 mm non-essential amino acids, 50 μm β-mercaptoethanol, and 15% heat-inactivated FBS. All cell lines tested negative for mycoplasma contamination. Cells were transfected with plasmids using Lipofectamine 2000 reagent according to the instructions of the manufacturer (Invitrogen).

##### Xenopus Experiments

*Xenopus* embryo collection, microinjection, whole-mount *in situ* hybridization, animal cap assays, and *papc* quantitative RT-PCR conditions have been described previously (27, 28). Riboprobes against *Xenopus ptk7* and *papc* have been described previously (9, 27). Antisense morpholino oligonucleotides (Gene Tools LLC) have been described previously: Ptk7 MOs (9, 12) and Ror2 MO (25). Synthetic capped mRNAs were produced with the Ambion (Applied Biosystems) mMessage mMachine kit. *Xenopus PTK7-FL-Venus* and *Xenopus PTK7-ICD-Venus* fusions were cloned into the pSpE3 vector, and capped mRNAs were synthesized with T3 polymerase after plasmid linearization with SfiI. For *Wnt5a*- and *mRFP*-capped RNA, *Wnt5a* in pCS2+ (provided by H. Steinbeisser) and *mRFP* in pCS2+ were linearized with Not1 and transcribed with Sp6. For immunofluorescence staining, whole gastrula embryos were blocked in 15% serum and incubated with anti-Venus and anti-RFP antibodies overnight at 4 °C, followed by 90-min incubation in Alexa Fluor 568 (anti-mouse) and Alexa Fluor 488 (anti-chick) fluorophore-conjugated antibodies. The injected ectoderm was explanted and mounted in Fluoromount for confocal analysis, and imaging was performed using a Zeiss LSM 780 microscope.

##### Knockdown Experiments

The ROR2 siRNA sequences used were as follows: ROR2 siRNA1, 5′-GCAA T G T GC T AG T G T ACGA TT-3′; ROR2 siRNA2, 5′-TAAAGGGTCGTTCGGATCCAGAACC-3′. Non-targeting siRNA controls were used (Life Technologies). Transfection with siRNAs was carried out with RNAiMAX (Invitrogen) as recommended by the supplier.

##### Antibodies and Recombinant Proteins

Monoclonal rat and polyclonal rabbit antibodies to PTK7 (1G9 and KN) were generated in the laboratory. Other antibodies used in this study according to the recommendations of the manufacturers were as follows: mouse antibody to α-tubulin (Sigma, catalog no. B512), rabbit antibody to Thr-183/Tyr-185 SAPK (stress-activated protein kinase)/JNK (Cell Signaling Technology, catalog no. 9251), polyclonal rabbit antibody to JNK (Santa Cruz Biotechnology, catalog no. sc-571), monoclonal mouse antibody to FLAG (Sigma, catalog no. F3165), monoclonal mouse antibody to MYC (Santa Cruz Biotechnology, catalog no. 9E10), monoclonal mouse antibody to HA (Covance, catalog no. MMS-101R-500), and secondary antibodies coupled to horseradish peroxidase (Jackson ImmunoResearch Laboratories). Recombinant human WNT5A was purchased from R&D Systems (catalog no. 645-WN-010).

##### Western Blots and Immunoprecipitation

Cells were lysed in lysis buffer (50 mm Hepes, 150 mm NaCl, 1 mm EDTA, 1 mm EGTA, 10% glycerol, 1% Triton X-100, 25 mm NaF, and 10 μm ZnCl2) supplemented with 0.5 mm PMSF, 1 mm orthovanadate, 1 mm β-glycerophosphate, and a protease inhibitor mixture (Sigma-Aldrich). For immunoprecipitation, after preclearing with agarose beads and incubation with antibodies, protein G-agarose beads were added to the lysates, and bound immune complexes were recovered and washed three times in lysis buffer. Proteins were resolved by SDS-PAGE, transferred to nitrocellulose filters, blocked for 1 h at room temperature in Tris-buffered saline/5% nonfat dry milk/0.1% Tween 20, and blotted overnight with primary antibodies in blocking solution. After extensive washings in TBS/0.1% Tween 20, filters were incubated for 1 h at room temperature with an HRP-conjugated secondary antibody before being revealed with an enhanced chemiluminescence substrate (West Pico, Thermo Scientific). Acquisition was performed with a G-BOX imager (Ozyme).

##### AP-1-responsive Firefly Luciferase Assays

HEK 293T cells expressing *Renilla* luciferase were seeded in 48-well plates and cultured until 80% confluence. Cells were co-transfected with an AP1-responsive firefly luciferase construct (Qiagen, AP1 reporter (luc) kit, catalog no. CCS-011L) plus expression vector and starved overnight. Then cells were stimulated with DMEM and 1% FCS with or without WNT5A. Luciferase expression was measured using Dual-Luciferase® reporter assay system protocol (Promega) and a Centro LB 960 microplate luminometer (Berthold Technologies).

##### Wound Healing Assays

Cells were seeded in 6-well plates precoated with rat tail collagen I and cultured on collagen I until confluence. Then cells were starved overnight and wounded using a pipette tip. Three wounds were made for each sample. Cells were stimulated with DMEM and 1% FCS with or without WNT5A. Cell migration was followed using video microscopy for 8 h, and analysis was performed with Metamorph software (Molecular Devices).

## Results and Discussion

### 

#### 

##### PTK7 Binds to ROR2 and WNT5A

**FIGURE 1.**
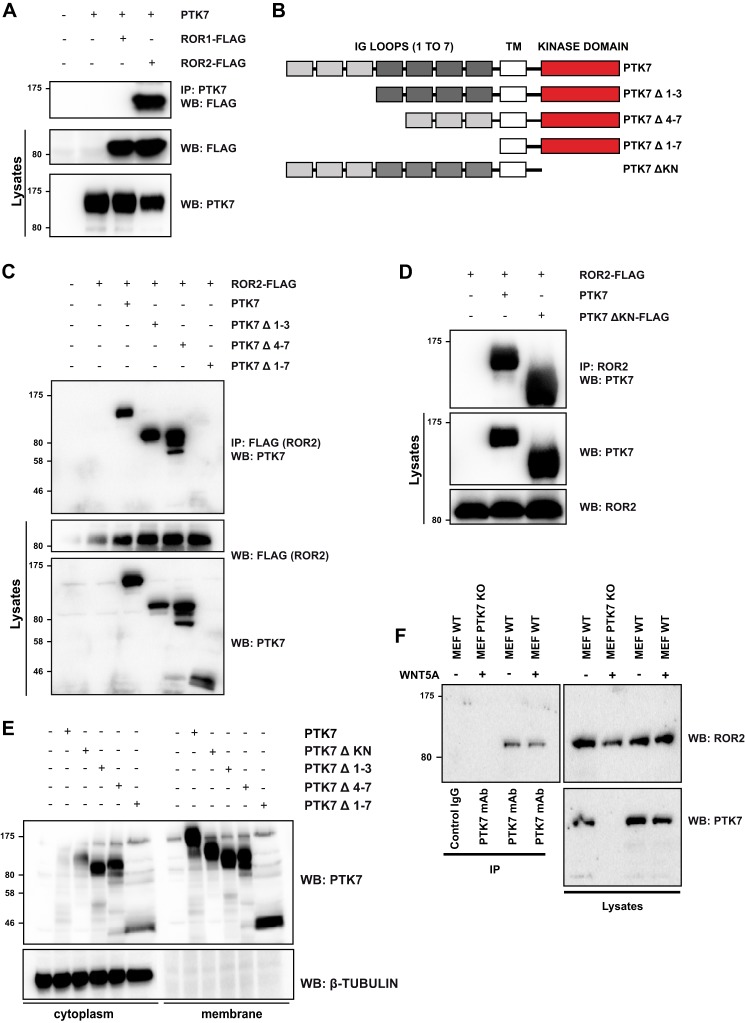
**PTK7 interacts with ROR2.** *A*, PTK7, FLAG-ROR1 and FLAG-ROR2 were co-expressed in HEK 293T cells. Proteins extracted from cell lysates were immunoprecipitated (*IP*) with anti-PTK7 antibody and revealed with the mentioned antibodies. *WB*, Western blot. *B*, schematic of PTK7 constructs used in this study. *TM*, transmembrane domain. *C* and *D*, FLAG-ROR2 was co-expressed in HEK 293T cells with the constructs expressing the truncated forms of PTK7, and co-immunoprecipitation was done as in *A. E*, cytosol/membrane fractionation of HEK 293T cells expressing the truncated forms of PTK7. Cell lysates were obtained using hypotonic lysis buffer. Samples were centrifuged to obtain cytosolic fractions. Membrane fractions were obtained by ultracentrifugation (1 h, 40,000 rpm) of the cytosolic fraction. β-Tubulin was used as a cytosolic control. *F*, proteins extracted from MEFs stimulated with WNT5A (200 ng/ml) or left unstimulated were subjected to co-immunoprecipitation with anti-PTK7 (PTK7) antibody or an isotype-matched control antibody (IgG). After Western blot analysis, total cell lysates and immunoprecipitated proteins were probed with the mentioned antibodies.

PTK7 has been shown to heterodimerize with VEGFR1 (19), an endothelial RTK. Here we hypothesized that ROR2, another active RTK implicated in PTK7-like developmental processes, may act as a candidate co-receptor for PTK7 in other cell types. ROR2 belongs to an RTK family comprising another homologous receptor, ROR1 (24). To evaluate the interaction between PTK7 and ROR family members, co-immunoprecipitation between ectopically expressed ROR1, ROR2, and PTK7 was performed in HEK 293T cells. We found that PTK7 formed a complex with ROR2 but not with ROR1 (Fig. 1*A*). To determine the region of PTK7 involved in the binding to ROR2, we generated a series of PTK7 mutants lacking extracellular Ig-like or intracellular regions (Fig. 1*B*). In co-immunoprecipitation assays, we showed that deletion of the whole extracellular region, but not of the intracellular region, inhibited the interaction with ROR2. The entire extracellular region of PTK7 was apparently required because deleted forms (PTK7Δ1–3 and Δ4–7) could still co-immunoprecipitate with ROR2 (Fig. 1, *C* and *D*). Using a cell fractionation procedure, we showed that all PTK7 mutants were present in the membrane fractions (Fig. 1*E*).

**FIGURE 2.**
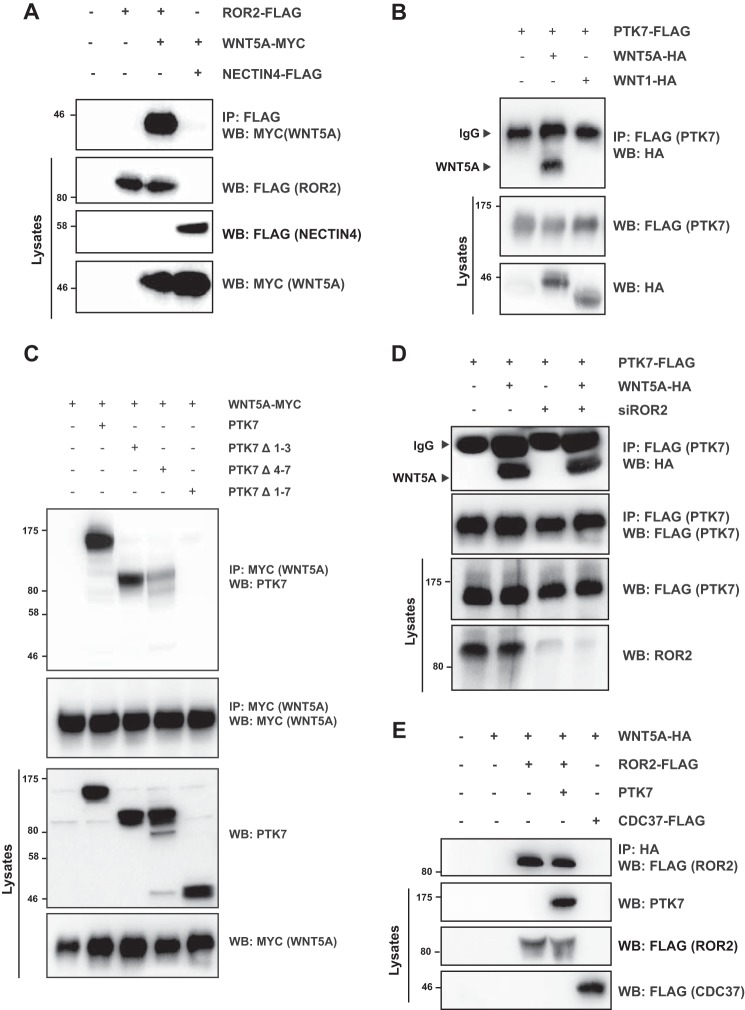
**PTK7 interacts with WNT5A.** *A*, FLAG-ROR2 and MYC-WNT5A were co-expressed in HEK 293T cells. Cell lysates were subjected to co-immunoprecipitation (*IP*) with anti-FLAG antibody and then submitted to Western blot (*WB*) analysis with the mentioned antibodies. FLAG-NECTIN4 was used as a negative control. *B*, FLAG-PTK7 was co-expressed with HA-WNT1 or HA-WNT5A in HEK 293T cells. Co-immunoprecipitations were done as in *A. C*, full and truncated isoforms of PTK7 were co-expressed with MYC-tagged WNT5A in HEK 293T cells. Cell lysates were subjected to co-immunoprecipitation with anti-MYC antibody and then submitted to Western blot analysis with the mentioned antibodies. *D*, PTK7-FLAG and HA-WNT5A were co-expressed in HEK 293T cells transfected with a siRNA directed against ROR2 or left untransfected. Cell lysates were subjected to co-immunoprecipitation with anti-FLAG antibody followed by Western blot analysis. *E*, PTK7, FLAG-ROR2, and HA-WNT5A were co-expressed in HEK 293T cells. Cell lysates were subjected to co-immunoprecipitation with anti-HA antibody, followed by Western blot analysis. FLAG-CDC37 was used as a negative control.

We confirmed the PTK7-ROR2 interaction at the endogenous level in MEFs expressing or not expressing Ptk7 (Fig. 1*F*). Together, these data identify ROR2 as a receptor able to associate with PTK7. ROR2 has been described as a cell surface receptor for non-canonical WNT ligands such as WNT5A (24). Indeed, ROR2 and WNT5A could be co-immunoprecipitated upon ectopic expression in HEK 293T cells (Fig. 2*A*). To investigate a potential interaction between PTK7 and WNT5A, PTK7 was co-expressed with HA-tagged WNT5A or WNT1 in HEK 293T cells, and lysates were prepared for co-immunoprecipitation. As shown in Fig. 2*B*, PTK7 efficiently interacted with WNT5A but not WNT1. The presence of the entire extracellular region of PTK7 was required for the interaction with WNT5A (Fig. 2*C*). Deletion of the Ig-like loops (Δ1–3) or (Δ4–7) did not abolish the binding, although a weaker interaction was found between WNT5A and PTK7Δ4–7 (Fig. 2*C*). To evaluate whether WNT5A binding to PTK7 was indirect and due to PTK7-ROR2 heterodimerization, we depleted ROR2 with a specific siRNA and repeated the PTK7-WNT5A co-immunoprecipitation (Fig. 2*D*). The absence of ROR2 did not impair the interaction between WNT5A and PTK7, and overexpression of PTK7 did not modify the amount of ROR2 co-immunoprecipitated with WNT5A (Fig. 2*E*). Moreover, WNT5A stimulation did not affect the PTK7-ROR2 interaction, suggesting that these receptors bind independently to WNT5A (Fig. 1*F*). Taken together, these results show that PTK7 can form a complex with ROR2 and WNT5A in mammalian cells through its extracellular region. Loops 4–7 in PTK7 are apparently required for optimal binding to WNT5A but not ROR2.

##### PTK7 Participates in WNTA Signaling and Cell Movements in a JNK-dependent Manner

**FIGURE 3.**
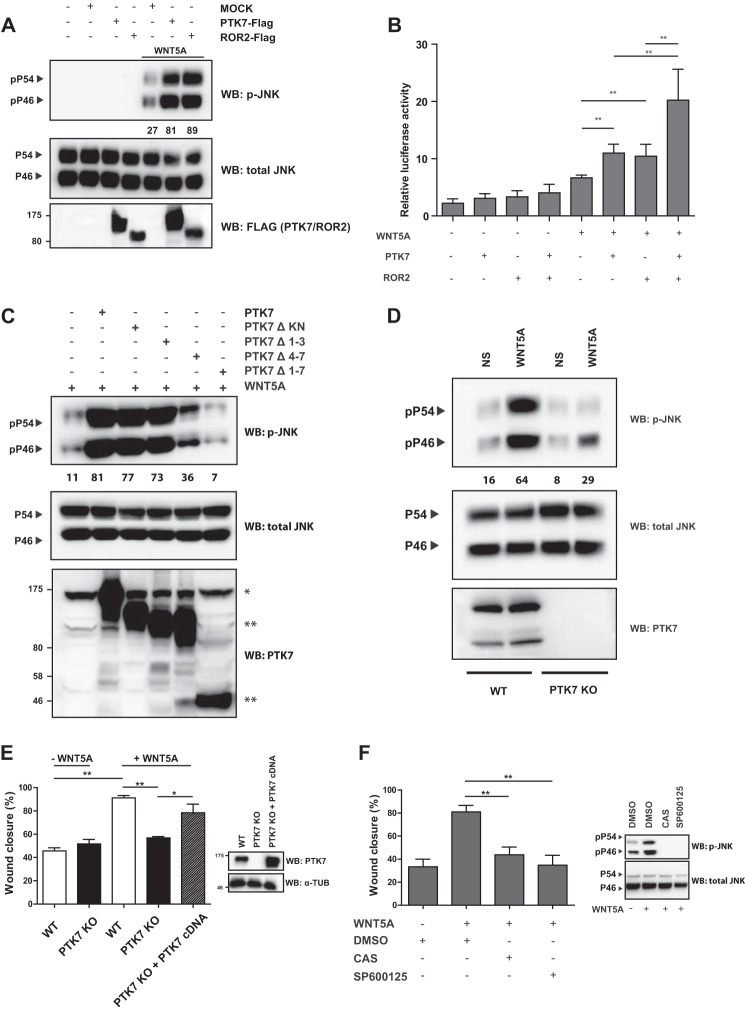
**WNT5A utilizes the PTK7-JNK pathway to promote cells movements.** *A*, FLAG-PTK7 and FLAG-ROR2 were overexpressed in HEK 293T cells. After 8-h starvation, cells were stimulated for 15 min with WNT5A (200 ng/ml). Cell lysates were subjected to Western blot (*WB*) analysis with the mentioned antibodies. *pP54* and *P54* refer to the phosphorylated and non-phosphorylated p54 JNK isoform, respectively. *pP46* and *P46* refer to the phosphorylated and non-phosphorylated p46 JNK isoform, respectively. *B*, FLAG-tagged PTK7 and ROR2 were co-expressed with an AP-1 luciferase reporter in HEK 293T cells, and luciferase activity was measured as detailed under “Experimental Procedures.” Data are representative of three experiments. Significant difference was determined by Student's *t* test. **, *p* < 0.01. *C*, various constructs expressing full-length or truncated forms of PTK7 (*two asterisks*) were overexpressed in HEK 293T cells. After 8-h starvation, cells were stimulated for 15 min with WNT5A (200 ng/ml). Cell lysates were subjected to Western blot analysis with the mentioned antibodies. Endogenous PTK7 is indicated by *one asterisk. D*, MEFs isolated from WT or gene-trapped *ptk7* (PTK7 KO) mice were starved for 8 h and stimulated for 15 min with WNT5A (200 ng/ml). Cell lysates were subjected to Western blot analysis with the mentioned antibodies. *NS*, nonstimulated. *E*, MEFs, WT or PTK7 KO, were seeded in 6-well plates, starved, and incubated with DMEM and 1% FCS with or without WNT5A (200 ng/ml) and submitted to wound healing. The percentage of wound closure was evaluated after 8 h. Re-expression of PTK7 (*right panel*, Western blot of protein extracts using the mentioned antibodies) was able to partially rescue the loss of cell migration in PTK7 KO cells. Data are representative of three experiments. Significant difference was determined by Student's *t* test. *, *p* < 0.05; **, *p* < 0.01. α*-TUB*, α-tubulin. *F*, JNK activity is required for WNT5A-induced wound healing in MEFs. The experiment was performed as shown in *E*, except that MEFs were treated with two JNK inhibitors: CAS 129-56-6 (*CAS*, 100 μm) and SP600125 (100 μm) (*left panel*). *Right panel*, lysates of MEFs were probed with the indicated antibodies. *DMSO*, dimethyl sulfoxide.

To further analyze the contribution of PTK7 to WNT5A-induced signaling, we examined the phosphorylation of JNK, a downstream effector of the WNT5A/ROR2 pathway (29). In HEK 293T cells, expression of ROR2 or PTK7 alone did not lead to JNK phosphorylation. However, stimulation of each receptor with WNT5A induced a comparable and robust phosphorylation of the p54 and p46 JNK isoforms (Fig. 3*A*). Phosphorylated JNK activates the c-JUN protein, which is known to form the activator protein 1 (AP-1) transcription factor. We used an AP-1-responsive firefly luciferase construct to monitor the transcriptional activity of AP-1 in HEK 293T cells expressing PTK7 or ROR2. Upon WNT5A stimulation, ROR2 and PTK7 similarly induced AP-1-dependent gene transcription that correlated to JNK phosphorylation. Co-expression of both receptors led to an additive effect on AP-1 reporter activation (Fig. 3*B*). From these data, we concluded that, like ROR2, PTK7 responds to WNT5A by inducing a JNK cascade in mammalian cells. Interestingly, in experiments using the truncated forms of PTK7, we found that deletion of loops 4–7 decreased JNK phosphorylation upon WNT5A stimulation (Fig. 3*C*). These data are in agreement with binding data showing weaker binding of WNT5A to the PTK7 Δ4–7 mutant (Fig. 2*C*). We next aimed to assess the role of the WNT5A-PTK7-JNK pathway at the functional level. We used primary MEFs because these cells express endogenous PTK7 (Fig. 1*F*) and have been used to characterize the WNT5A/ROR2/JNK cascade for its involvement in cell motility (30). We first looked at the level of JNK phosphorylation upon WNT5A stimulation in WT and PTK7-deficient (PTK7 KO) MEFs. We observed robust JNK phosphorylation after WNT5A treatment (Fig. 3*D*), which was impaired significantly (50% decrease) in the absence of PTK7. These data confirmed that PTK7 is implicated, in part, in WNT5A signal transduction in MEFs. We next used a wound healing assay to evaluate the contribution of the WNT5A-PTK7-JNK pathway to cell movements. Unstimulated wild-type MEFs closed 40% of the wound in 8 h, whereas addition of WNT5A led to an almost (90%) complete closure (Fig. 3*E*). In contrast, PTK7-deficient MEFs were not responsive to WNT5A and presented a similar closure with or without ligand. Importantly, we could rescue WNT5A-induced cell migration by re-expressing PTK7 in PTK7-deficient MEFs (PTK7 KO + PTK7 cDNA). To investigate the contribution of JNK activity in PTK7-induced cell movements, wild-type MEFs were treated with two different JNK inhibitors (CAS 129-56-6 and SP600125) or dimethyl sulfoxide and assayed in wound healing experiments as in Fig. 3*E*. WNT5A-induced cell motility of wild-type MEFs was abolished by JNK inhibition compared with the control condition (Fig. 3*F*). We concluded that PTK7 promotes cell movements in primary MEFs upon WNT5A stimulation by activating a JNK-dependent signaling pathway. These data are very comparable with those showing that WNT5A triggers a ROR2-JNK signaling pathway to regulate MEF cell migration (30). Therefore, both receptors bind and respond to WNT5A. However, despite the expression of ROR2 in MEFs (data not shown), which allows a partial phosphorylation of JNK upon WNT5A stimulation (Fig. 3*D*), the presence of PTK7 is required for WNT5A activity in wound healing assays (Fig. 3*E*).

##### Functional Interaction between PTK7 and ROR2 in Xenopus

**FIGURE 4.**
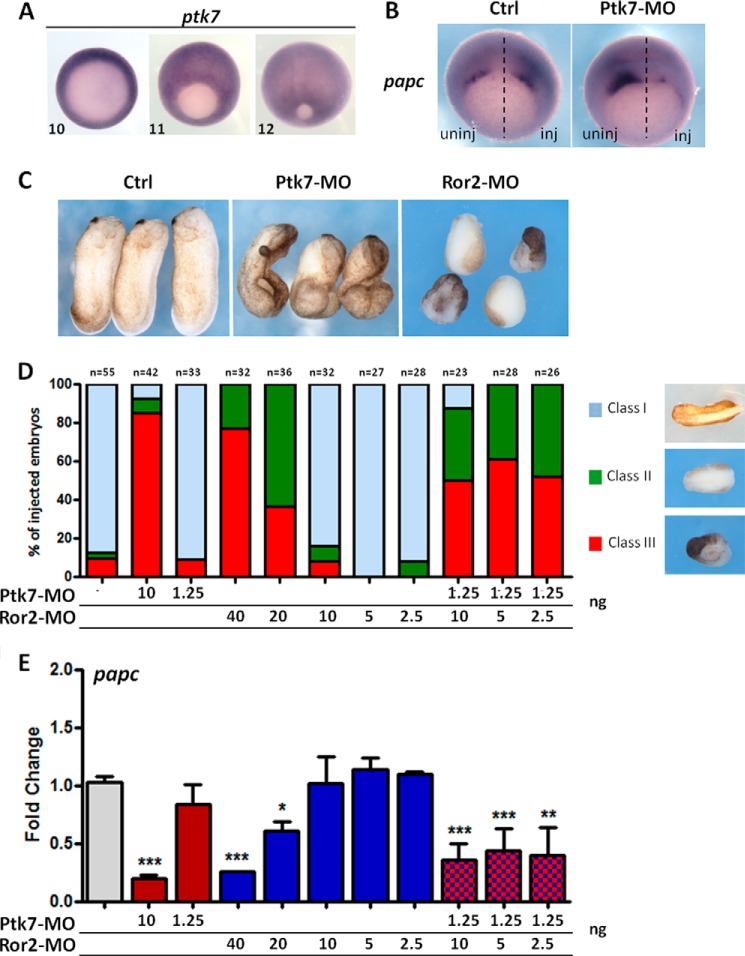
**Ptk7 cooperates with Ror2 during *Xenopus* embryo morphogenesis.** *A*, whole-mount *in situ* hybridization of *ptk7* in early (*stage 10*), mid- (*stage 11*), and late (*stage 12*) gastrula embryos. *B*, two-cell embryos were injected (*inj*) into one single blastomere with 10 ng of Ptk7-MO and processed for whole-mount *in situ* hybridization of *papc* at mid-gastrula stage. *Uninj*, uninjected; *Ctrl*, control. *C*, two-cell embryos were injected into each blastomere with 10 ng of Ptk7-MO or 40 ng of Ror2-MO. Morphology was analyzed at tail bud stage. *D*, two-cell embryos were injected in each blastomere with the indicated amounts of Ptk7-MO and Ror2-MO. Morphology was analyzed at tail bud stage. The number of injected embryos is indicated above the columns. Class I embryos are morphologically normal, class II embryos are shorter and have a wider neural plate, and class III embryos show severe neural tube closure defects. *E*, embryos injected as in *D* were collected at late gastrula (*stage 13*) and processed for quantitative RT-PCR. For all quantitative PCR graphs, *error bars* represent mean ± S.E. of three independent experiments with two technical duplicates. For statistical analyses, samples were compared with the respective control using unpaired Student's *t* test. *, *p* < 0.05; **, *p* < 0.005; ***, *p* < 0.005.

During *Xenopus* development, the Wnt5a/Ror2 pathway regulates embryonic morphogenesis through induction of the key downstream effector *papc* (also known as *pcdh8*) in the involuting mesoderm (25). On the basis of the above data, we hypothesized that *Ptk7* could be involved in the activation of *papc* by the Wnt5A/Ror2 pathway. Consistent with this possibility, *Ptk7* transcripts were detectable in the ectoderm and in the involuting mesoderm during gastrulation (Fig. 4*A*). To test our hypothesis, we first injected Ptk7 antisense morpholinos (Ptk7-MO) (9). This led to a dramatic decrease of *papc* expression on the injected side of gastrula embryos (Fig. 4*B*). To further test a possible functional interaction between Ptk7 and Ror2, we injected Ptk7-MO and Ror2-MO (25) separately or together. When injected separately at high doses, both Ptk7-MO and Ror2-MO injection severely interfered with embryonic morphogenesis (Fig. 4*C*) and repressed *papc* expression, as measured by quantitative RT-PCR (Fig. 4*E*). In contrast, development proceeded normally, and *papc* expression was maintained when suboptimal amounts of Ptk7-MO or Ror2-MO were injected (Fig. 4, *D* and *E*). However, co-injection of suboptimal amounts of both MO led to severe morphogenesis defects, and *papc* was repressed significantly (Fig. 4, *D* and *E*). Together, these data support the existence of a functional interaction between PTK7 and ROR2 in *Xenopus* that is required for correct morphogenesis.

**FIGURE 5.**
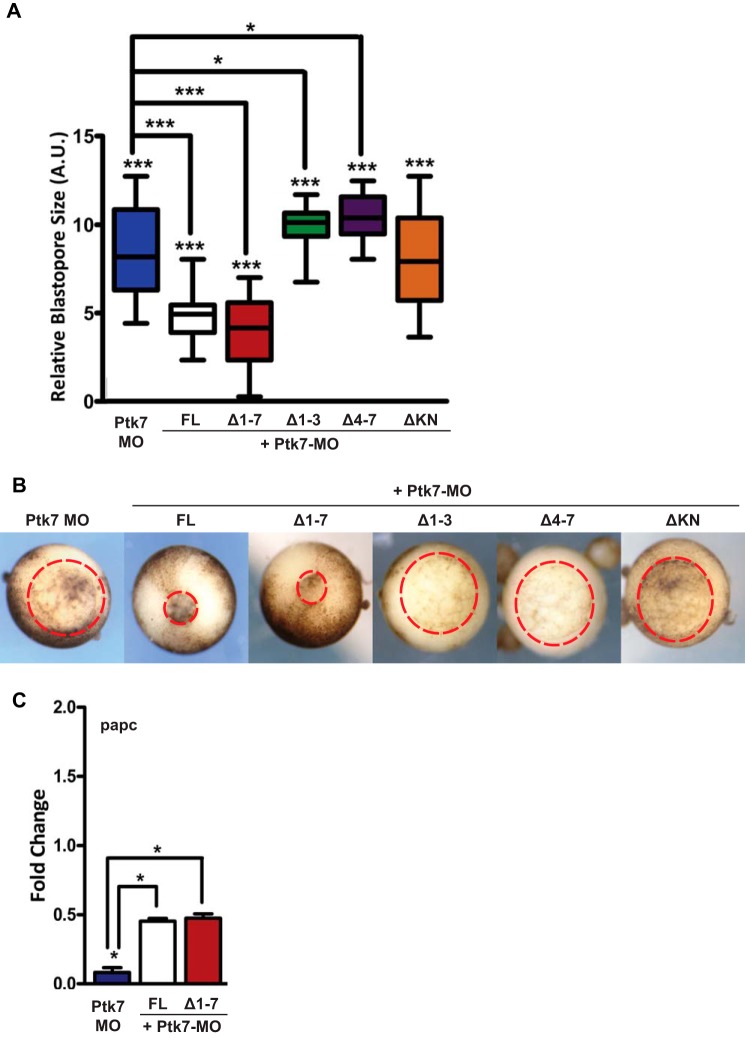
**Rescue assays of Ptk7 morphant phenotypes by human PTK7 constructs.** *A* and *B*, two-cell embryos were injected in each blastomere with 10 ng of Ptk7 MO, followed by injection at the four-cell stage of synthetic transcripts (200 pg total for each RNA on the basis of initial dose-response tests) encoding the various human PTK7 constructs used in this study (see Fig. 1*B*). *A*, blastopore closure was estimated using the ratio of blastopore diameter to the mean of control blastopore diameter at stage 13. *Bars* represent maximum and minimum values, and the *line* represents the mean. 15–27 embryos/condition were used for the analysis. *A.U.*, arbitrary units. *B*, representative blastopore closure phenotypes recorded (vegetal views, the blastopore is delineated in *red*). Note that only full-length (*FL*) human PTK7 and Δ1–7 PTK7 could significantly rescue blastopore closure of Ptk7 morphant embryos. *C*, embryos injected as in *A* were processed for quantitative RT-PCR at stage 13 for *papc* expression. Full-length human PTK7 and Δ1–7 PTK7 could significantly reactivate *papc* expression in morphant embryos. For statistical analysis of blastopore closure, samples were compared with the respective control (*asterisk over the column*) and Ptk7-MO (*asterisk over the line*) using unpaired Student's *t* test. For the quantitative PCR graph, *error bars* represent mean ± S.E. of two independent experiments with two technical duplicates. For quantitative RT-PCR statistical analysis, samples were compared with the respective control (*asterisk over the column*) and Ptk7-MO (*asterisk over the line*) using unpaired Student's *t* test. *, *p* < 0.05; **, *p* < 0.005; ***, *p* < 0.005.

We next decided to test the potential conservation of PTK7 function between humans and *Xenopus*. We performed rescue assays of Ptk7 morphants with the human PTK7 constructs used as shown in Fig. 1*B*. Using the recovery of blastopore closure as a readout, we obtained significant rescue with full-length human PTK7 and with the Δ1–7 deletion construct but not with other mutant forms (Fig. 5, *A* and *B*). The rescue with full-length PTK7 indicates that the human and frog counterparts share the same biochemical activity and that functional conservation exists between species. The lack of rescue with Δ1–3, Δ4–7, and ΔKN deletion constructs implies that both extracellular and intracellular domains of PTK7 are important and that these constructs are likely unable to interact with the natural PTK7 partners. Of interest, the potent rescue with Δ1–7 human PTK7 suggests that perhaps it acts through the intracellular domain of PTK7 (PTK7-ICD) (15, 18). In addition, *papc* expression was rescued in Ptk7 morphants by re-expressing full-length human PTK7 and the PTK7 Δ1–7 construct (Fig. 5*C*).

**FIGURE 6.**
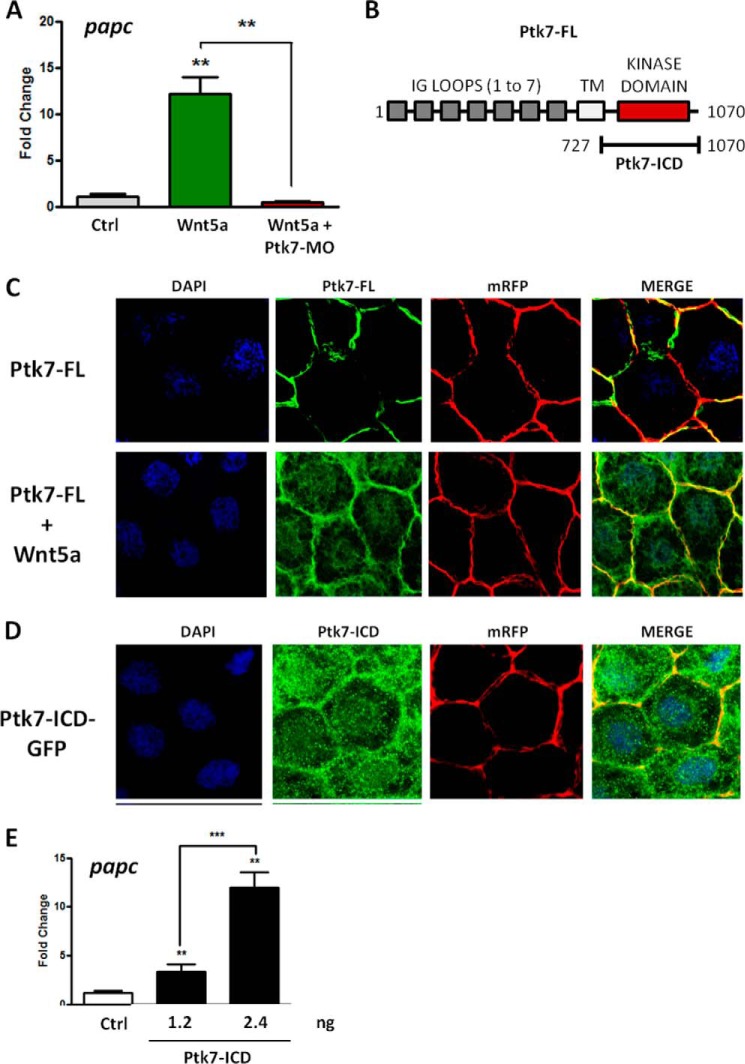
**Ptk7 is required for Wnt5A-mediated *papc* induction.** *A*, four-cell embryos injected with *Wnt5a* mRNA (30 pg/cell) in the animal pole received a second injection of Ptk7-MO (2. 5 ng/cell) in all animal blastomeres at the eight-cell stage. Animal caps were isolated at blastula stage, cultured for 4 h at 23 °C, and then processed for quantitative RT-PCR. *Ctrl*, control. *B*, schematic of *Xenopus* full-length PTK7 and PTK7-ICD constructs. GFP was added at the C terminus to generate the fusions used in experiments *C–E. TM*, transmembrane domain. *C*, eight-cell embryos were injected with full-length *Ptk7-GFP* mRNA (250 pg/cell) and *mRFP* mRNA (50 pg/cell) to reveal cell membranes, with or without *Wnt5a* mRNA (30 pg/cell) in all animal blastomeres. Embryos were grown until early gastrula stage (stage 10.5) and processed for confocal imaging. PTK7-GFP localized strictly to cell membranes in the absence of Wnt5a and partly relocalized to the nucleus in the presence of Wnt5a. *D*, eight-cell embryos were co-injected with *Ptk7-ICD-GFP* mRNA (300 pg/cell) and *mRFP* mRNA (50 pg/cell) in all animal blastomeres. Embryos were grown until early gastrula stage (stage 10.5) and processed for confocal imaging. PTK7-ICD-GFP was found at the cell membrane and in the nucleus. *E*, eight-cell embryos were injected with the indicated amounts of *PTK7-ICD-GFP* mRNA in all animal blastomeres, and animal caps were isolated at blastula stage, cultured for 4 h at 23 °C, and then processed for quantitative RT-PCR. For all quantitative PCR graphs, *error bars* represent mean ± S.E. of three independent experiments with two technical duplicates. For statistical analyses, samples were compared with the respective control using unpaired Student's *t* test. **, *p* < 0.005; ***, *p* < 0.005.

Next we examined more directly whether Ptk7 was required for Wnt5a-induced *papc* expression (25). Injection of *Wnt5a* RNA in naïve ectoderm induced a massive induction of *papc* expression that was totally suppressed in the presence of Ptk7-MO (Fig. 6*A*). It has been shown that the PTK7 receptor is subjected to proteolytic cleavage in cancer cell lines and that the released PTK7-ICD is able to translocate into the nucleus (15, 18) We hypothesized that, in *Xenopus* cells, Wnt5A could trigger the cleavage and translocation of Ptk7-ICD. As expected, injection into naïve ectoderm of a construct encoding a C-terminal fusion of GFP with full-length Ptk7 revealed a strict localization at the cell membrane (Fig. 6, *B* and *C*). However, when this construct was expressed together with *Wnt5a* RNA, we could detect the GFP signal in the membrane, in the cytoplasm, and in the nucleus (Fig. 6*C*). This result was consistent with the release of Ptk7-ICD induced by Wnt5A but could also reflect changes in the trafficking of full-length Ptk7. Therefore, we evaluated the subcellular localization of a truncated construct encoding Ptk7-ICD fused to GFP at its C terminus. We found that, upon injection of this construct into naïve ectoderm, the GFP signal was also distributed between the plasma membrane, the cytoplasm, and the nucleus (Fig. 6*D*). Importantly, quantitative RT-PCR analysis revealed that Ptk7-ICD, in the absence of exogenous Wnt5a, was capable of strongly activating *papc* expression in a dose-dependent manner (Fig. 6*E*). Our data suggest that, beyond its interaction with Ror2, Ptk7 may be cleaved when bound to Wnt5a and may directly participate in transcriptional activation of downstream targets such as *papc*.

Our report highlights novel findings regarding the role of PTK7 in WNT/PCP signaling. First, we provide compelling evidence of a physical interaction between PTK7 and ROR2, a WNT/PCP-related receptor. Binding is specific in the ROR family and occurs through the entire PTK7 extracellular domain (Fig. 1). Second, in contrast to a report published previously (11), we find that WNT5A readily co-immunoprecipitates with PTK7 and, like ROR2, induces JNK phosphorylation and cell movements (Figs. 1 and 3). We cannot explain the lack of interaction between WNT5A and PTK7 reported by Peradziryi *et al.* (11). The recipients used for the co-immunoprecipitation experiments are, however, different between this study and ours. In the cited report, co-immunoprecipitations were performed in *Xenopus* egg extracts, whereas ours were done in HEK 293T cells. However, we controlled the functionality of our WNT5A construct by showing its interaction with ROR2, a well known interactor (Fig. 2*A*). In Peradziryi *et al.* (11), the authors proposed that, by binding to canonical WNT3A or WNT8 ligands, PTK7 inhibited canonical WNT activity by sequestering these ligands. Another report has shown that WNT5A inhibited the canonical WNT pathway by promoting the degradation of β-catenin (31). Our findings suggest that PTK7 can directly trigger the non-canonical WNT signaling pathway through its binding to WNT5A. Binding could be direct or indirect through Frizzled receptors, as shown for WNT3a (11), and is partially dependent on loops 4–7 (Fig. 2*C*). Third, we evidence a functional interaction between Ptk7 and Ror2 in *Xenopus* and a dependence of Wnt5a to Ptk7 (Fig. 4). Fourth, using rescue assays in *Xenopus*, we determined that human and frog PTK7 have conserved functions (Fig. 5). However, compared with our assays in HEK 293T cells, the situation is more complicated in *Xenopus* because both extracellular and intracellular regions of PTK7 are required for the function of the receptor, in particular its ICD. Indeed, we provide evidence for a physiological function of Ptk7 ICD (Fig. 6), which, added to data obtained in cancer cells, suggest that cleavage and nuclearization are important processes for Ptk7 activity (15, 17, 18). Accordingly, aberrant proteolysis of Ptk7 has a profound effect on embryonic development (32). However, under the conditions of our assay, Ror2 appeared to be dispensable for the release and nuclear translocation of PTK7 ICD in *Xenopus* (data not shown). Future studies will have to assess how PTK7 and ROR2 cross-talk within the heterodimeric complex at the signaling and functional levels.

## Author Contributions

S. M. and A. C. L. carried out experiments in mammalian cells and wrote the experimental part of the paper. M. G. made all human PTK7 constructs. A. M. D. generated stable HEK 293T clones allowing AP-1 luciferase assays and shRNA constructs. V. T. made the *Xenopus* Ptk7 constructs. P. S. carried out all experimental procedures in *Xenopus*. L. K. and J. P. B conceived the project and wrote the manuscript.

## Footnotes

5 The abbreviations used are:

PCP: planar cell polarity
RTK: tyrosine kinase receptor
MEF: mouse embryonic fibroblast
MO: morpholino oligonucleotide
ICD: intracellular domain.
